# Plasma biomarkers in a cohort of cases with dementia and in cognitively preserved nonagenarians

**DOI:** 10.1002/alz.086078

**Published:** 2025-01-09

**Authors:** Estrella Gómez‐Tortosa, Pablo Agüero, Alicia Ruiz Gonzales, Sonia Wagner, Ignacio Mahillo, Andrea Ruiz, María José Sainz, Raquel Téllez, Lucía Cremades‐Jimeno, Pascual Sanchez‐Juan

**Affiliations:** ^1^ Fundación Jiménez Díaz, Madrid, madrid Spain; ^2^ Alzheimer´s Centre Reina Sofía‐CIEN Foundation‐ISCIII, Madrid, madrid Spain; ^3^ Neurology Department, Hospital Universitario Marqués de Valdecilla – IDIVAL – University of Cantabria ‐ CIBERNED, Madrid Spain

## Abstract

**Background:**

Recent reports support the use of plasma biomarkers of neurodegeneration and neuroinflammation, as determined through ultrasensitive single molecular arrays (SIMOA), to screen and diagnose patients with dementia. However, their translation to clinical settings requires further studies.

**Methods:**

We evaluated plasma samples from 186 individuals including 72 patients with AD (supported by CSF biomarkers consistent with an A+T+N+ classification scheme), 44 with confirmed FTD, 48 cognitively intact nonagenarians, and 22 controls (ages 40‐83 years). We measured AD core biomarkers (Abeta40, Abeta42, total tau and p‐tau181), neurofilament light chain (NFL), and glial fibrillary acid protein (GFAP) using SIMOA with Quanterix assays. In cases with dementia we correlated AD plasma biomarkers with those in CSF, obtained by Lumipulse G600II immunoassay. We calculated the best plasma cutoff values for each biomarker to discriminate among AD, FTD and cognitively preserved individuals.

**Results:**

As regards AD core biomarkers, AD cases showed lower Abeta42/Abeta40 ratio as compared to FTD cases and controls (p<0.001), and higher p‐tau (p=0.06 vs FTD, p=0.007 vs controls). These two biomarkers also showed a significant correlation with CSF values (Spearman p>0.001). GFAP was significantly elevated in dementia cases and nonagenarians as compared to controls with the following gradient: nonagenarians (median=344) > AD (314)> FTD (225); the same occurs for NFL with the following gradient: nonagenarians (median=41)> FTD (39.4) > AD (20). In the distinction between FTD and AD, plasma NFL showed better accuracy (AUC 0.80) than Abeta42/Abeta40 ratio (AUC 0.76), GFAP (AUC 0.68), and p‐tau (AUC 0.66). In cognitively preserved nonagenarians Abeta42 was significantly higher than in AD cases (p=0.001) and controls (p=0.01), tau and p‐tau was higher than controls but with no significant differences, while both NFL and GFAP were in the highest values of the four groups.

**Conclusions:**

In this single‐center clinical cohort we confirm the different patterns of plasma AD core biomarkers in AD and FTD cases, as well as an opposite pattern of elevation regarding NFL and GFAP. Cognitively preserved nonagenarians have positive degeneration and inflammation biomarkers supporting resilience rather than resistance to AD.

